# Global Soil Hydraulic Properties dataset based on legacy site observations and robust parameterization

**DOI:** 10.1038/s41597-022-01481-5

**Published:** 2022-07-25

**Authors:** Surya Gupta, Andreas Papritz, Peter Lehmann, Tomislav Hengl, Sara Bonetti, Dani Or

**Affiliations:** 1 Soil and Terrestrial Environmental Physics, Department of Environmental Systems Science, ETH Zürich, Zürich, Switzerland; 2 OpenGeoHub Foundation, Wageningen, the Netherlands; 3 EnvirometriX, Wageningen, the Netherlands; 4 Soil Physics and Land Management Group, Wageningen University, Wageningen, The Netherlands; 5 Division of Hydrologic Sciences, Desert Research Institute, Reno, NV USA

**Keywords:** Hydrology, Environmental sciences

## Abstract

The representation of land surface processes in hydrological and climatic models critically depends on the soil water characteristics curve (SWCC) that defines the plant availability and water storage in the vadose zone. Despite the availability of SWCC datasets in the literature, significant efforts are required to harmonize reported data before SWCC parameters can be determined and implemented in modeling applications. In this work, a total of 15,259 SWCCs from 2,702 sites were assembled from published literature, harmonized, and quality-checked. The assembled SWCC data provide a global soil hydraulic properties (GSHP) database. Parameters of the van Genuchten (vG) SWCC model were estimated from the data using the R package ‘*soilhypfit*’. In many cases, information on the wet- or dry-end of the SWCC measurements were missing, and we used pedotransfer functions (PTFs) to estimate saturated and residual water contents. The new database quantifies the differences of SWCCs across climatic regions and can be used to create global maps of soil hydraulic properties.

## Abstract (summary)

**Table:** 

Measurement(s) Soil water content at their given matric potential
Technology Type(s) pressure plate • sandbox apparatus • pressure chamber

## Background & Summary

The soil water characteristics curves (SWCCs) describes the relationship between soil water content (in gravimetric or volumetric form) and matric potential^1,2^ and is a fundamental soil hydraulic property that characterizes soil water dynamics and key hydrological processes^3^. The amount of water retained in the soil pores at given matric potential is dominantly controlled by soil texture, structure, and organic matter^4^. The measurement of the SWCCs in the laboratory or field is laborious and time-consuming^5^. This limitation hinders empirical characterization of SWCC parameters over large areas as required in land surface and catchment-scale modeling. An alternative to excessive sampling to obtain spatially distributed SWCC parameters is the application of pedotransfer functions (PTFs)^6^. However, agronomic legacy of soil mapping influences PTF-derived SWCCs, which tend to be region-specific and focus on homogeneous agricultural soil^7–9^. The growing demand for hydrological land surface parameterization beyond agricultural lands and the related refinement of spatial representation require more definitive SWCC information^10^. Motivated by the growing need for more comprehensive and spatially resolved SWCCs, a key objective of this study was to pool published datasets and supplement these with anecdotal literature values for regions with poor coverage. A cursory inspection of major datasets such as WOSIS^11^, UNSODA^12^ and measurements by Holtan^13^ shows critical deficiencies ranging from lack of spatial referencing to partial information with datasets omitting the wet-end (water content measured for matric potential ≤0.2 m) and dry-end (water content measured at matric potential ≥150 m) that is often defined as wilting point of SWCCs. For example, WOSIS^11^ and Holtan^13^ datasets provide SWCCs starting at 0.6 and 1.0 m matric potential, respectively, and missing the wet-end measurements. In contrast, the ZALF dataset^14^ provides wet-end information but dry-end information is missing. In addition, Batjes *et al*.^11^ and Holtan^13^ used bulk density measured at 3.3 m matric potential (defined as density at field capacity) to convert gravimetric to volumetric water content, ignoring the water mass retained in the pore space at field capacity. Therefore, a critical aspect of compiling a global SWCCs dataset for hydro-climatic applications is to harmonize the data and devise strategies for imputing missing information as we further elaborate in the section *‘Materials and methods’*.

The objectives of this study are to address the limitations of currently available datasets (such as lack of wet- and dry-end measurements of SWCCs, poor estimates of dry bulk density, etc.) and to provide a systematic approach to infer more reliable and geo-referenced SWCC parameters. We put an effort to collect a global soil hydraulic properties (GSHP) database with 15,259 SWCCs by importing, quality controlling, and standardizing tabular data from existing datasets and scientific literature and estimating van Genuchten (vG) model parameters from the measured SWCCs. For the quality control, firstly, we excluded SWCCs using likelihood-based confidence intervals, yielding 11,705 as ‘good quality estimate’. Along with SWCCs, GSHP also provides information regarding soil texture, bulk density, organic carbon, and porosity. The database also contains 8,675 data of Ksat that allow the quantification of the unsaturated hydraulic conductivity function. The GSHP database covers most countries, climatic regions, and continents, including tropical regions with more intense soil-forming processes and inactive clay minerals. The data and codes are made publicly available to promote data-driven analysis and to collect additional data to increase the accuracy of global modeling of unsaturated soil properties in land surface and Earth system models.

## Methods

### Data sources

The GSHP database was assembled in two steps. Firstly, we combined well-known datasets such as UNSODA^12^, HYBRAS^15^, WOSIS^11^, and AFSPDB^16^. Secondly, we used different search engines, including Science Direct (https://www.sciencedirect.com/), Google Scholar (https://scholar.google.com/) and Scopus (https://www.scopus.com) to collect additional data. We searched for SWCC datasets using *“soil water retention curves”*, *“moisture retention curves”*, and *“soil water characteristics curves”* as keywords. Moreover, the country names were also used with these keywords to conduct more specific searches for areas with few or no data points (this was the case, for example, for Cambodia, Vietnam, and Thailand). The sources of the collected datasets are listed in Table 1 together with the number of SWCCs for each dataset.

**Table 1:** List of sources for SWCC data and number of SWCCs (*N*) per dataset assembled in the GSHP database.

Reference	*N*	Reference	*N*	Reference	*N*
Al-Darby and El-Shafei^42^	1	Li *et al*.^43^	3	Al Majou*et al*.^44^	10
Alghamdi *et al*.^45^	1	Abid and Lal^46^.	4	Asghari*et al*.^47^	12
Are *et al*.^48^	1	Bescansa *et al*.^49^	4	MacVicar *et al*.^50^	12
Babaeian *et al*.^51^	1	Dlapa *et al*.^52^	4	Noguchi *et al*.^53^	12
Bhushan and Sharma^54^	1	Hoshino *et al*.^55^	4	Tobón *et al*.^56^	12
de Oliveira *et al*.^57^	1	Kumar *et al*.^58^	4	Tyagi *et al*.^59^	14
Garba *et al*.^60^	1	McBeath *et al*.^61^	4	AL-Kayssi^62^	15
Glab *et al*.^63^	1	Mondal *et al*.^64^	4	Karup *et al*.^65^	16
Kakeh *et al*.^66^	1	Ng *et al*.^67^	4	Simmons^68^	16
Lowe *et al*.^69^	1	Smettem and Gregory^70^	4	Wang *et al*.^71^	16
Medina *et al*.^72^	1	Xia *et al*.^73^	4	Cooper *et al*.^74^	18
Nyamangara *et al*.^75^	1	Chari and Vahidi^76^	5	Quang and Jansson^77^	20
Sulaeman *et al*.^78^	1	Eden *et al*.^79^	5	Pan *et al*.^80^	22
Thakur *et al*.^81^	1	Moazeni-Noghondar *et al*.^82^	5	Bambra^83^	23
Wickland *et al*.^84^	1	Nano *et al*.^85^	5	Marui *et al*.^86^	25
Zebarth *et al*.^87^	1	Toriyama *et al*.^88^	5	Vereecken and Van Looy^89^	145
Zhang *et al*.^90^	1	Xing *et al*.^91^	5	Jauhiainen *et al*.^92^	108
El-Asswad *et al*.^93^	2	Jha *et al*.^94^	6	Richard and Lüscher^36^	111
Ismail^95^	2	Konyai *et al*.^96^	6	Forrest *et al*.^17^	115
Khdair *et al*.^97^	2	Li *et al*.^98^	6	Vereecken and Van Looy^89^	145
Lozano *et al*.^99^	2	Li *et al*.^100^	6	Kool *et al*.^101^	217
Macinnis-Ng *et al*.^102^	2	Manyame *et al*.^103^	6	Nemes *et al*.^12^	218
Mosquera *et al*.^104^	2	Talat *et al*.^105^	6	CSIRO^106^	652
Mujdeci *et al*.^107^	2	Ullah *et al*.^108^	6	Leenaars *et al*.^16^	729
Abedi-koupai *et al*.^109^	3	Werisch *et al*.^110^	6	Ottoni *et al*.^15^	814
Basile and D’Urso^111^	3	Elliott and Price^112^	7	Stolbovoy *et al*.^113^	1,129
Cuenca *et al*.^114^	3	Ismail^115^	8	Holtan^13^	1,864
De Boever *et al*.^116^	3	Novak^117^	8	Batjes *et al*.^11^	2,541
Guzman *et al*.^118^	3	Saha and Kukal^119^	8	Grunwald^19^	6,008
Kassaye *et al*.^120^	3	Seki *et al*.^121^	8		

The next task was to check the availability of spatial coordinates for the measurements in each dataset. We assigned each SWCC to one of eight *‘accuracy classes’* ranging from highest (0–100 m) to lowest (more than 10,000 m or non-available information (NA)) accuracy. For example, Forrest *et al*.^17^, and Ottoni *et al*.^15^ provided exact coordinates of the locations, thus we assigned a location accuracy of 0–100 m (i.e., highly accurate; see Table S1 for more details). For other references, we digitized provided maps or sketches with locations of the measurements. We first georeferenced these maps using ESRI ArcGIS software (v10.3) and then digitized the coordinates from georeferenced images. Some of the documents we digitized (e.g. Nemes *et al*.^12^) provided the names of specific locations, and hence we used Google Earth to obtain the coordinates. Coordinates of SWCCs for which only a location name was provided, were estimated using Google Earth, while SWCCs with neither coordinates nor location name were discarded. A detailed description of the extraction of coordinates is provided in Gupta *et al*.^18^. Note that Batjes *et al*.^11^, and Leenaars *et al*.^16^ used another definition of accuracy classes in their collection of datasets. Therefore, we merged the various accuracy definitions into broader classes as shown in Table S1. For example, samples that have minimum and maximum location accuracy less than 100 m (likely 0–10 m, 0–40 m, etc) are assigned to the 0–100 m class. Likewise, 0–1000 m values are assigned to the 500–1000 m class. Furthermore, datasets were cross-checked to avoid redundancy. For example, the WOSIS dataset^11^ includes the AFSPDB dataset^16^, so we removed the redundant data from WOSIS and used the original dataset (AFSPDB) in the final compilation. The Florida dataset^19^ was directly obtained from a project website.

### Database cleaning and harmonization

The harmonization of the collected datasets was done by first converting all data to the same units. The units are described in Table 2. Note that we used potential heads (unit of length) and not pressure values for the characterization of the water potential. Furthermore, the data was cleaned as follows: a) SWCCs with maximum volumetric water content more than 1.0 m^3^/m^3^ were removed, b) SWCCs that had less than four data pairs were removed, c) SWCCs in which the water content increased more than 10% for increasing (absolute) matric potential were removed (see Figure S1), and d) SWCCs without wet-end information that did not have bulk density data were removed because it was impossible to impute the saturated water content without such information.

**Table 2:** List of all 54 variables in the GSHP database and their units.

Header	Description	Units
layer_id	Unique ID of each SWCC	—
disturbed_undisturbed	Sample soil structure disturbed or undisturbed during the analysis	—
climate_classes	Climate information (temperate, boreal etc.)	—
profile_id	Unique ID of each profile	—
reference	Data reference	—
DOIs_URLs	Data DOIs or URLs	—
method	Method used to measure the SWCC	—
method_keywords	Comments on the methods if applicable	—
latitude_decimal_degrees	Ranges up to +90 degrees down to −90 degrees	Decimal degree
longitude_decimal_degrees	Ranges up to + 180 degrees down to −180 degrees	Decimal degree
hzn_desgn	Soil horizon designation	—
hzn_top	Upper depth of soil sample	cm
hzn_bot	Lower depth of soil sample	cm
db_33	Bulk density at 3.3 m matric potential	g/cm^3^
db_od	Dry bulk density	g/cm^3^
oc	Soil organic carbon content	%
tex_psda	Soil texture classes based on USDA	—
sand_tot_psa	Mass of soil particle 2 mm for fine earth	%
silt_tot_psa	Mass of soil particle > 0.05 and < 2 mm for fine earth	%
clay_tot_psa	Mass of soil particles < 0.002 mm for fine earth	%
ph_h2o	Soil reaction	—
ksat_field	Soil saturated hydraulic conductivity from field	cm/day
ksat_lab	Soil saturated hydraulic conductivity from lab	cm/day
porosity	Porosity	m^3^/m^3^
WG_33kpa	Gravimetric water content at 3.3 m matric potential	kg/kg
lab_head_m	Lab measured matric potential	m
lab_wrc	Lab measured volumetric water content	m^3^/m^3^
field_head_m	Field measured matric potential	m
field_wrc	Field measured volumetric water content	m^3^/m^3^
keywords_total_porosity	Extra information regarding porosity	—
SWCC_classes	SWCC classes (indicators for presence of wet- and dry-end information)	—
source_db	Source of the data	—
location_accuracy_min	Minimum value of location accuracy	m
location_accuracy_max	Maximum value of location accuracy	m
broad_accuracy_classes	Classes for location accuracy	—
*α*	vG shape parameter	m^−1^
se_*α*	Standard error of *α* vG shape parameter	m^−1^
*n*	vG shape parameter	—
se_*n*	Standard error of *n* vG shape parameter	—
*θ*_*r*_	Residual water content	m^3^/m^3^
*θ*_*s*_	Saturated water content	m^3^/m^3^
q2.5_*α*	2.5^*th*^ percentile of *α*	m^−1^
q97.5_*α*	97.5^*th*^ percentile of *α*	m^−1^
q10_*α*	10^*th*^ percentile of *α*	m^−1^
q90_*α*	90^*th*^ percentile of *α*	m^−1^
q25_*α*	25^*th*^ percentile of *α*	m^−1^
q75_*α*	75^*th*^ percentile of *α*	m^−1^
q2.5_*n*	2.5^*th*^ percentile of *n*	—
q97.5_*n*	97.5^*th*^ percentile of *n*	—
q10_*n*	10^*th*^ percentile of *n*	—
q90_*n*	90^*th*^ percentile of *n*	—
q25_*n*	25^*th*^ percentile of *n*	—
q75_*n*	75^*th*^ percentile of *n*	—
data_flag	Classes that defines the quality of the vG parameters	—

After data extraction from literature, geo-referencing, and harmonization, all information was collected in tabular form in the new GSHP database. The database consists of 54 variables (see Table 2) and 136,989 records with water content measurements (and complementary information) at given matric potential recorded for 15,259 SWCCs. A list of the variables of the database along with their units is given in Table 2.

### Conversion of gravimetric to volumetric water content

Some datasets such as Holten^13^ and Batjes *et al*.^11^ provided the gravimetric water content, and the dry bulk density (ratio of solid mass to total volume), before potential shrinkage was required to convert to volumetric water content. However, for these samples, the dry bulk density of a soil clod had been determined by measuring the volume (and mass) of a dry ‘clod’ of soil and not by measuring the soil sample volume at wet state (for shrinking soils, the dry bulk density will be overestimated by measuring the sample volume at dry state). For these datasets, the ‘wet’ bulk density at 3.0 or 3.3 m matric potential had been measured as well, and this value had been used in the original analysis to convert gravimetric to volumetric water content. However, this ‘wet’ bulk density (including water mass) is higher than the dry bulk density. Here we followed a different strategy for conversion to volumetric water content and assumed that the volume of the ‘clod’ of soil at 3.3 m matric potential is the same as at saturation. This is a simplification because soils with structural pores can shrink by application of −3.3 m matric potential as observed by Assi *et al*.^20^ and Bonvin *et al*.^21^. However, the reported bulk density changes between 0.1 and 5.2% (average 3%) were small compared to the error when using bulk density measured at 3.3 m matric potential to convert gravimetric to volumetric water mass (typically, about 20% of sample mass is water at a matric potential of −3.3 m). The adapted expression for dry bulk density is then equal to:

1 $${\rho }_{bulk}=\frac{{m}_{solid}}{{V}_{total}}=\frac{{\rho }_{3.3m}}{\left(1+{\theta }_{grav3.3m}\right)}$$

where *ρ*_*bulk*_ is the dry bulk density, *m*_*solid*_ is the mass of solid particles, *V*_*total*_ is the volume of the soil clod at 3.3 m matric potential), *ρ*_3.3 *m*_ is the bulk density and *θ*_*grav*3.3 *m*_ is the gravimetric water content at 3.3 m matric potential. Note that Holten^13^ provided the gravimetric water content at 3 m matric potential, but we assumed for simplicity that the water contents at 3 and 3.3 m matric potential are equal.

### PTFs for constraining saturated (*θ*_*s*_) and residual (*θ*_*r*_) water contents for SWCCs without wet- and dry-end measurements

In this study, SWCCs were modeled using van Genuchten (vG) Eq. 2.

The vG model provides the volumetric water content, *θ* (m^3^/m^3^) at matric potential *ψ* (m) as

2 $$\theta (\psi )={\theta }_{r}+({\theta }_{s}-{\theta }_{r}){[1+{(\alpha | \psi | )}^{n}]}^{-(1-1/n)}$$

where *θ*_*s*_ and *θ*_*r*_ are the saturated and residual water contents, respectively (m^3^/m^3^), and *α* (m^−1^) and *n* (dimensionless) are SWCC shape parameters.

To estimate the vG parameters, measurements close to full and residual saturation are needed. The GSHP database contains some SWCCs that lack the wet- and/or dry-end information (i.e., no measurements are available at matric potential ≤ 0.2 m or ≥ 150 m, respectively). Fitting the vG model to SWCCs without wet-end information leads to unreliable parameter estimates as shown in Figure S2 and Table S2. Therefore, PTFs were used to impute the wet- and/or dry-end information in this study.

#### PTF for *θ*_*s*_

A PTF for *θ*_*s*_ was developed and tested based on those SWCCs that had water content information at matric potentials ranging from 0.00 or 0.01 m (saturated water content) to 150 m (permanent wilting point). In total, 2,287 SWCCs had both wet- and dry-end measurements and from this set we selected those 1,947 SWCCs with soil texture and bulk density data to build the PTF. After fitting the vG model to these SWCCs see below, a robust linear regression PTF was developed for *θ*_*s*_ using the R package *‘robustbase’*^22^. This package is useful when the residual errors of the regression model have long-tailed distributions.

The linear regression PTF for *θ*_*s*_ was developed using, as covariates, clay and sand contents, bulk density, and a categorical variable that distinguished between tropical and other climatic regions (to account for unique soil-formation conditions in tropical climate). Two PTFs of *θ*_*s*_ were developed depending on the available covariate information: The first PTF (Model1) was developed for the SWCCs when both soil texture and bulk density were available and the second PTF (Model2) for the SWCCs when only bulk density information was available. To account for the intense weathering processes in the wet and warm climate of the tropical regions^23^, we distinguished between SWCCs from tropical and other climate regions (arid, boreal, temperate, and polar). Tropical regions have often Oxisols that are dominated by inactive (non-swelling) clay minerals (kaolinite) as shown by Ito and Wagai^24^ and are characterized by intense soil-formation processes affecting soil hydraulic properties^25^. The climate region information was extracted from the Köppen-Geiger climate zone map^26,27^.

The accuracy of the PTF was assessed by 20-fold cross-validation using coefficient of determination (*R*^2^), Root Mean Square Error (*RMSE*) and *BIAS* as accuracy measures. *BIAS* and *RMSE* are defined as: $BIAS={\sum }_{i=1}^{{N}_{1}}\frac{\left({\widehat{\theta }}_{s,i}-{\theta }_{s,i}\right)}{{N}_{1}}$ and $RMSE=\sqrt{\frac{{\rm{SSE}}}{{N}_{1}}}$ *where* $SSE={\sum }_{i=1}^{{N}_{1}}{\left({\widehat{\theta }}_{s,i}-{\theta }_{s,i}\right)}^{2}$. *SSE* is the sum of squared errors between the cross-validation predictions ${\widehat{\theta }}_{s,i}$ and the measurements *θ*_*s, i*_, and *N*_1_ is the total number of SWCCs. The coefficient of determination *R*^2^ is defined as: ${R}^{2}=\left[1-\frac{{\rm{SSE}}}{{\rm{SST}}}\right]$ *where* $SST={\sum }_{i=1}^{{N}_{1}}{\left({\theta }_{s,i}-{\bar{\theta }}_{s,i}\right)}^{2}$. SST is the total sum of squares and ${\bar{\theta }}_{s,i}$ is the arithmetic mean of the *θ*_*s*_ deduced from fitting the measured SWCCs. We calculated the prediction interval for *θ*_*s*_ at 95% confidence and used their upper and lower limits as box constraints for *θ*_*s*_ when estimating the vG parameters by the *‘soilhypfit’* ^28^ (https://cran.r-project.org/web/packages/soilhypfit/index.html) R package for the SWCCs lacking wet-end measurements.

#### Tuller and Or^29^ model for *θ*_*r*_

A physically-based model by Tuller and Or^29^ was used to constrain estimates of *θ*_*r*_ for the SWCCs where dry-end measurements were missing. The model describes the dry-end gravimetric water content (*θ*_*m*_) as a function of specific surface area (*SA*, in m^2^/kg) and the thickness of water film (*h*, in meters) adsorbed on mineral surfaces,

3 $${\theta }_{m}=h\cdot SA\cdot {\rho }_{w}$$

where *ρ*_*w*_ is the density of water. According to Or and Tuller^30^, capillary contribution becomes negligible for matric potential above 1000 m for a wide range of soil textures. For water adsorbed on planar surfaces by van der Waals forces, Iwamatsu and Horii^31^ expressed the equilibrium thickness (*h*) of an adsorbed thin water film as a function of a matric potential (*ψ*) as shown below in Eq. 4:

4 $$h=\sqrt[3]{\frac{{A}_{svl}}{6\pi g{\rho }_{w}\psi }}$$

where *A*_*svl*_ (J) is the Hamaker constant for solid-vapor interactions (we used *A*_*svl*_ equal to 6 × 10^−20^ J as proposed by Tuller and Or^29^), *ψ* (m) is the matric potential, *ρ*_*w*_ (kg/m^3^) is the density of the liquid, and *g* (m/s^2^) is acceleration due to gravity. According to Or and Tuller^32^, the water film thickness of an adsorbed water layer is equal to 3.5⋅10^−10^ m. At 150 m matric potential, the water thickness according to Eq. 4 is approximately 7.0⋅10^−10^ m (two layers of water molecules). For SWCCs without dry-end information, we constrained the possible range of residual water content using Eq. 3 with thickness *h* between one and two monolayers of water (3.5⋅10^−10^–7.0⋅10^−10^ m).

### Constraints on vG shape parameters

We constrained the possible values of *n* from 1.0 to 7.0. These limits were assigned considering literature, expert knowledge, and values obtained from fitting the SWCCs without constraints. We used minimum and maximum *α* values of 0 and 100 (m^−1^) for all textural classes. Because the inverse of *α* characterizes the capillary pressure in the largest soil pores, the maximum value imposed for *α* corresponds to a maximum pore diameter of 3 mm. Detailed Information on employed constraints when estimating the vG parameters is provided in Table S3.

### Fitting and quality check of the vG parameters using soilhypfit R package

SWCCs parameters were estimated, with box-constraints described in the previous sections, using the *‘soilhypfit’* R package^28^. *‘soilhypfit’* was designed for parametric modeling of SWCCs and/or unsaturated hydraulic conductivity datasets. The main function to estimate vG parameters is called *‘fit_wrc_hcc’* and uses maximum likelihood (ML) to estimate with the restriction *m* = 1 - 1/*n*. *‘fit_wrc_hcc’* uses optimisation algorithms of the NLopt library^33^ or the Shuffled Complex Evolution (SCE) algorithm^34^. Firstly, we used the default global optimisation algorithms to fit the SWCCs. Then, we refined the parameter estimates by the default unconstrained local algorithm, again fitting the untransformed parameters (*α*, *n*) with the same parameter range as in global optimization and using as initial values the parameters estimated by the global optimization algorithm. The reason for this sequential procedure is that the parameters returned by the global algorithm are sometimes slightly away from the parameters of the true maximum of the objective function and the computed standard errors of *α* and *n*, computed from the Hessian of loglikelihood function (=the observed Fisher information matrix), are then not accurate.

We checked the quality of estimates of the van Genuchten parameters *n* and *α* using loglikelihood profiles. From the profiles, we computed likelihood-based confidence intervals of *α* and *n* for 3 confidence levels (0.5, 0.8, and 0.95) (see chapter 4 in Uusipaikka^35^). Furthermore, we identified those SWCCs where the estimates of *α* and *n* coincided with any of the limits of the constraining intervals. We classified the parameter estimates in five classes based on the loglikelihood profiles and the standard errors of estimated parameter. The first two classes contained SWCCs with estimated parameters equal to the upper limits (*α* = 100, *n* = 7). For these SWCCs no standard error for the estimated parameters could be specified (classes denoted as ‘upper limit for *α*’ and ‘upper limit for *n*’). The third and fourth classes had flat likelihood profiles such that no quantiles of 75% probability or higher could be determined (classes denoted as ‘flat upper profile for *α*’ and ‘flat upper profile for *n*’). The fifth class or final class of the SWCCs was assigned to ‘good quality estimate’. After fitting the vG model to the SWCCs, standard deviations of the modelling errors, say ${\widehat{\sigma }}_{\theta }$ were computed and and SWCCs with ${\widehat{\sigma }}_{\theta }$ larger than 0.1 m^3^/m^3^ water content are discarded.

## Data Records

**Fig. 1:**
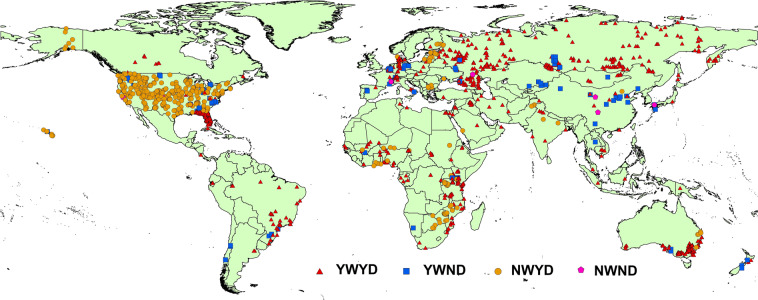
Spatial distribution of SWCCs in the GSHP database. A total of 2,702 locations are shown on the map. The locations are grouped into four classes: YW and NW stand for SWCCs with and without wet-end information (water content measured for matric potential ≤ 0.2 m), respectively, while YD and ND stand for SWCCs with and without dry-end information (water content measured at matric potential ≥ 150 m), respectively.

The database consists of 54 variables and 136,989 records with water content measurements at given matric potential (and complementary information) recorded for 15,259 SWCCs from 2,702 locations. A list of the variables of the database along with their units is given in Table 2. The global distribution of SWCCs is shown in Fig. 1, and the source of each dataset is given in Table 1. Table 3 shows the total number of SWCCs with the corresponding number *N* of θ-*ψ* data pairs from the various sources. The SWCCs with more than 5 data pairs dominate the database with 10,117 SWCCs followed by 3,784 and 1,359 SWCCs with 5 and 4 data pairs, respectively. The dataset with highest and lowest number of SWCCs were the Florida^19^ and the Swiss^36^ datasets, with 6,008 and 111 SWCCs, respectively. The ETH literature dataset designates the SWCC data that we collected by our own literature search. The database and readme file is uplodeded on the Zenodo platform and can be accessed using this link 10.5281/zenodo.5547338^37^.

**Table 3:** Number of SWCCs with 4, 5, and more than 5 data pairs (matric potential, water content) per sample along with a total number of SWCCs.

Source db	*N* = 4	*N* = 5	*N* > 5	Total number of SWCCs
AfSPDB^16^	186	255	288	**729**
Australian dataset^17,106^	1	119	648	**768**
ETH Literature	10	1884	747	**2641**
UNSODA^12^	2	2	214	**218**
WOSIS^11^	1159	374	1008	**2541**
HYBRAS^15^	—	11	803	**814**
Russia EGRPR^113^	—	1129	—	**1129**
Swiss dataset^36^	—	10	101	**111**
Belgium dataset^89^	—	—	145	**145**
Florida dataset^19^	—	—	6008	**6008**
ZALF dataset^14^	—	—	155	**155**
**Total**	**1,358**	**3,784**	**10,117**	**15,259**

The ETH literature dataset designates the SWCCs data that we collected by our own literature search, and it includes all other references shown in Table 1.

## Technical Validation

### Quality of PTFs for *θ*_*s*_ and *θ*_*r*_

**Fig. 2:**
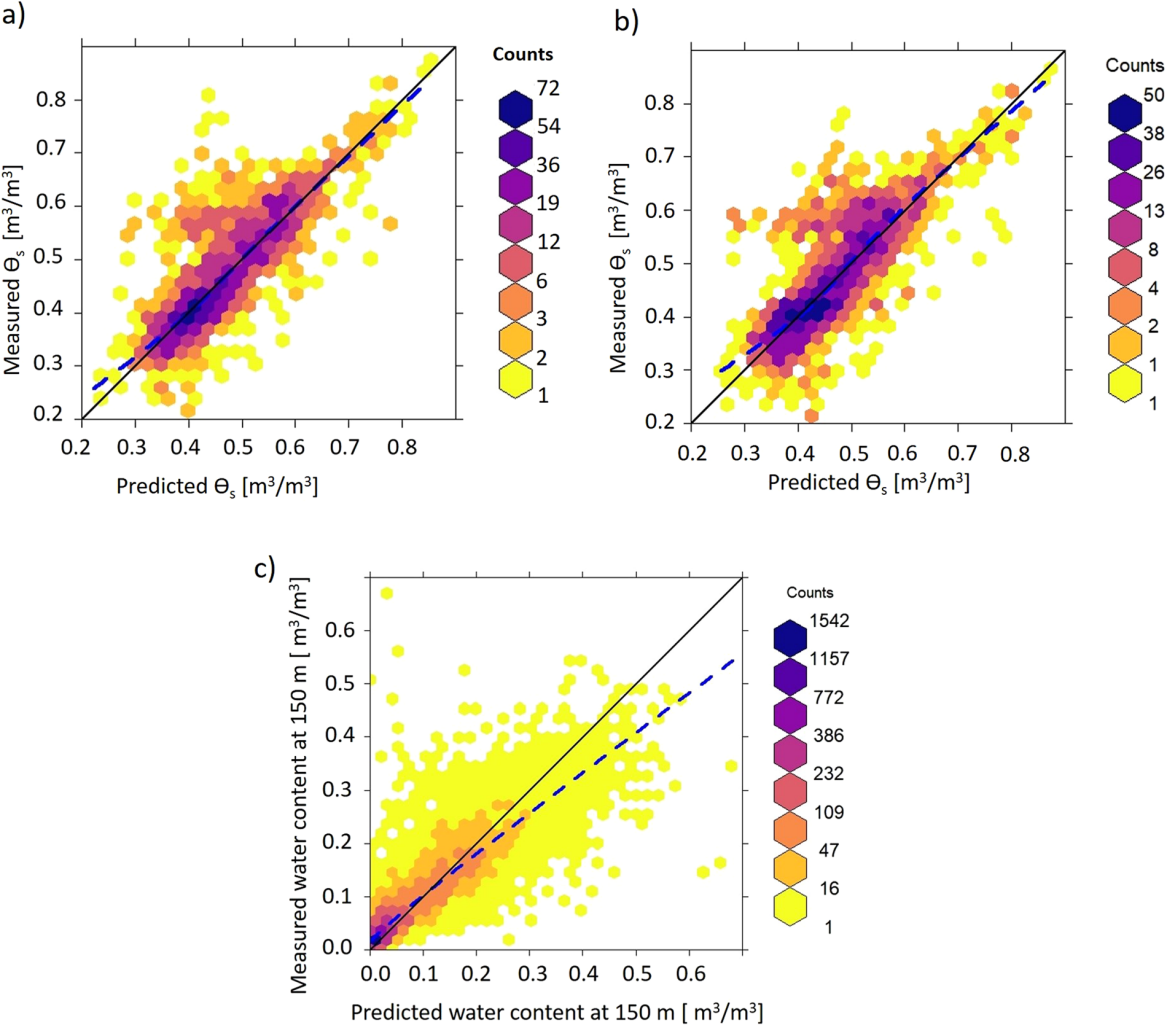
Performance of linear regression PTF to estimate *θ*_*s*_ and of the Tuller and Or^29^ model to estimate *θ*_*r*_ for SWCCs without wet- and dry-end information, respectively. (**a**) Relationship between measured values and cross-validation predictions of *θ*_*s*_ (measured *θ*_*s*_ are the values deduced from fitting the vG model to measured SWCCs). The solid black line is the 1:1 line, and the blue dashed line is the LOWESS (locally weighted scatter plot smoothing) curve. Cross-validation resulted in *R*^2^ = 0.645 and *RMSE* = 0.061 m^3^/m^3^ with *BIAS* = −0.009 m^3^/m^3^. (**b**) Performance of PTF that uses only bulk density to estimate *θ*_*s*_ for SWCCs without wet-end information measurements. Cross-validation resulted in *R*^2^ = 0.611 and *RMSE* = 0.066 m^3^/m^3^ with *BIAS* = −0.008 m^3^/m^3^. (**c**) Relation between measured and predicted water content at 150 m matric potential. Quantitative validation yielded the *R*^2^ = 0.752, *RMSE* = 0.053 m^3^/m^3^ with *BIAS* = −0.002 m^3^/m^3^.

Two PTFs for *θ*_*s*_ were developed for SWCCs without wet-end information (Eqs. 5–6). For 30% of all SWCCs, wet-end information was missing and had to be inferred from PTFs.

5 $${\theta }_{s}=a+b\cdot BD+c\cdot Clay+d\cdot Sand$$

6 $${\theta }_{s}=a+b\cdot BD$$

where *θ*_*s*_ (m^3^/m^3^) is the saturated water content, *BD* the bulk density (g/cm^3^) and *Sand* and *Clay* fractions are given in %. Note that we differentiated between tropical and other climatic regions. Only the values of the intercepts differ for tropical climate; coefficients *b*, *c*, and *d* are the same for all climatic regions. The coefficients are provided in Table 4. Moreover, the variance-covariance matrices and residual standard errors are provided for both models (Eqs. 5 and 6) in the supplementary document (Tables S4 and S5). The model was calibrated with 1,947 SWCCs, and 20-fold cross-validation was applied to validate the model. The results of 20-fold cross-validation for the model with bulk density and soil texture and differentiating between tropical and other climate regions are shown in Fig. 2a. Likewise, Fig. 2b shows the results for the model using only bulk density and climatic region. Moreover, the results for *θ*_*r*_ were validated by comparing the predicted water content at 150 m matric potential using the Tuller and Or model with the measured water content at 150 m as shown in Fig. 2c.

**Table 4:** Coefficients of linear regression PTFs for saturated water content *θ*_*s*_ for tropical and other climates.

Coefficients	Model1 (BD + clay + sand)		Model2 (BD)	
	Other climates	Tropical climate	Other climates	Tropical climate
*a*	0.917	0.932	0.987	1.011
*b*	−0.353	−0.353	−0.389	−0.389
*c*	0.00087	0.00087	—	—
*d*	−0.00004	−0.00004	—	—

### Spatial coverage and auxiliary soil properties

**Fig. 3:**
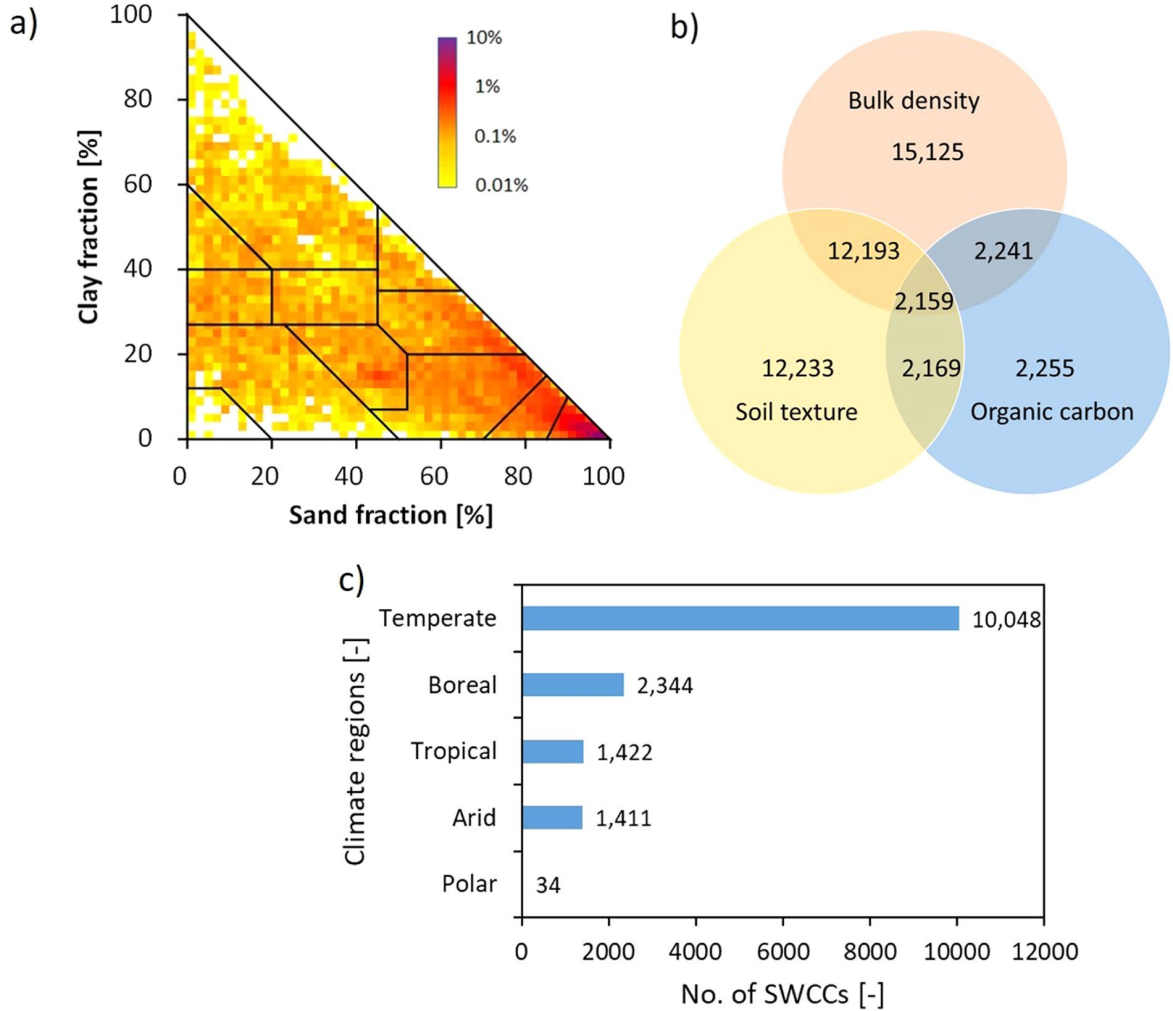
Overview of SWCC data. (**a**) Distribution of soil textures of samples in the GSHP database on the USDA soil texture triangle. (**b**) Venn diagram illustrating the number of SWCCs in the GSHP database for which bulk density, soil texture, and soil organic carbon data were also available. (**c**) Number of SWCCs per climatic regions.

The GSHP database contains SWCCs from all USDA soil textural classes (see Fig. 3a). Concerning the geographical distribution of the data, most of the SWCCs are from North America followed by Europe, Africa, Asia, South America, Australia/Oceania as shown in Table S6. 10,048 SWCCs belong to the temperate region, and 2,344, 1,422,1,411, and 34 SWCCs to boreal, tropical, arid, and polar regions, respectively (Fig. 3c). Regarding the measurement method of SWCCs, 99% of the SWCCs were measured in the laboratory and only 1% stem from the field (most field SWCCs are from the UNSODA dataset). Note that different laboratory methods were used to estimate SWCCs. Most commonly, pressure plate and sandbox apparatus for wet-end and pressure chamber for the dry-end measurements were used.

Along with SWCCs, in GSHP we also collected information on soil texture, bulk density, organic carbon, porosity, and saturated hydraulic conductivity (Ksat). Out of 15,259 SWCCs, 12,233, 15,125, 2,255, 2,754 SWCCs have information on soil texture, bulk density, organic carbon, porosity, respectively. In addition, 12,193 SWCCs have both soil texture and bulk density information, and 2,159 SWCCs have information on soil texture, bulk density, and organic carbon, as shown in Fig. 3b. Note that in addition to 12,233 soil texture values, 82 measurements have soil texture information with a total (sand + silt + clay) less than 98% or greater than 102%. We did not use these SWCCs in the development of the PTF for θ_s_, but kept them in the database and assigned the value “Error” to the soil texture class variable. The database also contains Ksat data for 8,675 soil samples that allow the quantification of the unsaturated hydraulic conductivity function using the Mualem-van Genuchten parameterization (see Figure S3). Regarding the number of soil profiles per location, in the GSHP database, 214 locations are without profile information whereas 2,390, 60, 17, and 21 locations have 1, 2–5, 6–10, and >10 soil profiles, respectively. Similarly, 259, 1,418, 916, and 109 locations have 1, 2–5, 6–10, and >10 soil samples, respectively. Moreover, 32 locations are without soil depth information whereas 287, 1,447, 876, 60 locations have 1, 2–5, 6–10, and >10 soil depths, respectively as shown in Table S7.

After the quality check of SWCCs, 10,373 SWCCs have been assigned as most accurate with a 0–100 m location accuracy. 1,890 SWCCs lack location accuracy information. Furthermore, after calculating the likelihood-based confidence intervals, a total of 11,705 SWCCs out of 15,259 emerges as ‘good quality estimate’ whereas 1,857, 633, 947, and 117 were classified as ‘flat upper profile for *α*’, ‘flat upper profile for *n*’, ‘upper limit for *α*’, and ‘upper limit for *n*’, respectively.

### Statistical characteristics of vG parameters

**Fig. 4:**
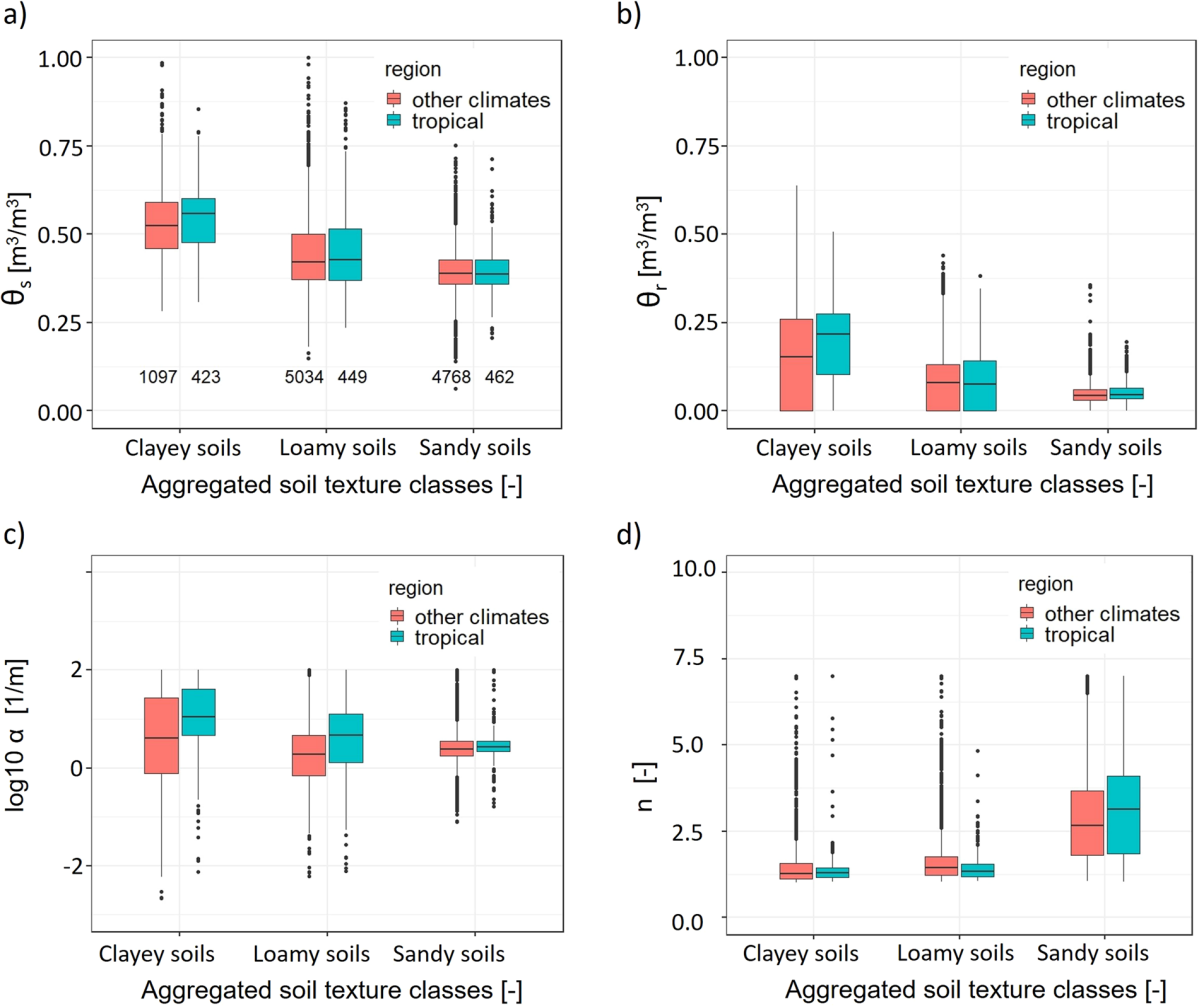
Distribution of vG parameters using aggregated soil texture classes (sandy soils: sand and loamy sand; loamy soils: sandy loam, loam, silt loam, silt, silty clay loam, clay loam, and sandy clay loam; clayey soils: sandy clay, silty clay, and clay) for SWCCs from tropical and other climatic regions. Note that soil textures are estimated using the USDA-Natural Resources Conservation Service soil texture triangle. The numbers in panel (**a**) show the number of vG parameters for different aggregated soil texture classes used to make this plots.

Table 5 reports the means and standard deviations of vG parameters and the number of SWCCs per soil textural class. The largest mean values of *α* were observed for clayey soils (classes clay and silty clay); loamy soils, and sandy soils showed smaller values of *α*. In contrast, the largest mean *n* was observed for sandy soils (classes loamy sand and sand). For the other textural classes, *n* ranged from 1.34 to 1.71. Similarly, highest and lowest *θ*_*s*_ and *θ*_*r*_ were observed for clayey and sandy soils, respectively. Regarding other soil properties, the largest Ksat values were obtained for sandy soils followed by clay soils, most likely due to the presence of macropores in these fine-textured soils^18^. Fig. 4 shows the distribution of vG parameters for combinations of aggregated soil texture classes and tropical and other climatic regions. In the database, there are 1,097 clayey, 5,034 loamy, and 4,768 sandy SWCCs from other climates and 423 clayey, 449 loamy, and 462 sandy SWCCs from tropical regions in the GSHP database.

**Table 5:** Means and standard deviations (format: mean, standard deviation) of basic soil properties, hydraulic conductivity, and vG SWCCs parameters per soil textural class.

Texture Classes	BD	OC	Porosity	Ksat	*α*	*n*	*θ*_*r*_	*θ*_*s*_
Clay	1.23, 0.22	1.34, 1.24	0.53, 0.08	4.00, 38.58	5.36, 8.98	1.59, 1.00	0.17, 0.13	0.55, 0.09
	(1,053)	(469)	(318)	(396)	(1,054)	(1,054)	(1,054)	(1,054)
Silty Clay	1.17, 0.25	1.79, 1.62	0.55, 0.08	1.25, 32.12	5.44, 12.43	1.47, 0.78	0.11, 0.11	0.52, 0.09
	(252)	(93)	(38)	(47)	(252)	(252)	(252)	(252)
Sandy Clay	1.44, 0.18	0.58, 0.80	0.43, 0.08	1.95, 19.05	4.97, 5.91	1.69, 0.93	0.17, 0.12	0.44, 0.08
	(214)	(61)	(40)	(132)	(214)	(214)	(214)	(214)
Clay Loam	1.27, 0.28	1.69, 2.23	0.56, 0.14	11.70, 28.96	3.10, 6.45	1.43, 0.59	0.09, 0.09	0.50, 0.12
	(427)	(104)	(44)	(70)	(428)	(428)	(428)	(428)
Silty Clay Loam	1.18, 0.24	1.69, 1.95	0.58, 0.12	2.9, 19.15	2.79, 6.49	1.49, 0.78	0.08, 0.08	0.52, 0.10
	(423)	(97)	(10)	(50)	(424)	(424)	(424)	(424)
Sandy Clay Loam	1.52, 0.20	0.60, 0.58	0.42, 0.07	1.09, 12.75	2.91, 4.29	1.63, 0.70	0.14, 0.10	0.41, 0.07
	(1061)	(200)	(80)	(703)	(1,062)	(1,062)	(1,062)	(1,062)
Silt	1.22, 0.21	1.44, 1.57	—	9.10, 7.81	0.65, 3.57	1.68, 0.46	0.03, 0.03	0.50, 0.07
	(36)	(18)	(0)	(10)	(36)	(36)	(36)	(36)
Silt Loam	1.24, 0.27	1.64, 2.48	0.51, 0.09	25.9, 9.40	1.30, 4.15	1.60, 0.58	0.06, 0.06	0.48, 0.10
	(849)	(259)	(19)	(193)	(857)	(857)	(857)	(857)
Loam	1.35, 0.29	1.36, 1.80	0.37, 0.08	15.54, 12.38	2.50, 4.65	1.50, 0.54	0.08, 0.07	0.46, 0.10
	(798)	(319)	(215)	(101)	(811)	(811)	(811)	(811)
Sandy Loam	1.46, 0.28	0.95, 1.42	0.42, 0.09	1.98, 10.34	1.88, 4.06	1.71, 0.65	0.08, 0.06	0.41, 0.09
	(1,858)	(316)	(56)	(789)	(1,865)	(1,865)	(1,865)	(1,865)
Loamy Sand	1.50, 0.21	0.55, 0.83	0.47, 0.06	5.11, 6.41	2.63, 3.44	1.90, 0.77	0.06, 0.04	0.39, 0.08
	(996)	(113)	(9)	(584)	(996)	(996)	(996)	(996)
Sand	1.50, 0.14	0.71, 1.00	0.43, 0.04	22.04, 3.34	2.66, 2.09	3.17, 1.34	0.04, 0.02	0.39, 0.06
	(4,226)	(120)	(17)	(3,884)	(4,234)	(4,234)	(4,234)	(4,234)
**Total number of SWCCs respective data**	**12,193**	**2,169**	**846**	**6,985**	**12,223**	**12,223**	**12,223**	**12,223**

The number of SWCCs is given in parenthesis. BD bulk density (g/cm^3^), OC soil organic carbon content (%), Porosity (m^3^/m^3^), Ksat saturated hydraulic conductivity (cm/day) measured in laboratory, *α* (m^−1^) and *n* (dimensionless) vG shape parameters, *θ*_*r*_ residual water content (m^3^/m^3^), *θ*_*s*_ saturated water content (m^3^/m^3^). For Ksat and *α* the geometric mean is reported (because Ksat and *α* are approximately lognormally distributed), while for all other properties, the arithmetic mean is provided.

## Usage Notes

### Challenges to determine vG parameters

The major problem to determine the vG parameters was missing measurements of water content close to saturation. Imposing the expected range of water content close to saturation had a large effect on the estimated vG parameters as shown with three illustrative examples in Figure S2 and Table S2. To scope with SWCCs that have not enough measured data pairs to cover the full matric potential and water content range, we imposed limits for the range of parameter values based on literature. Although we provided the PTF-based values of *θ*_*s*_ for SWCCs that lack the wet-end information, after estimating the likelihood-based confidence intervals, we noticed that some of the SWCCs are fitted with vG parameters equal to upper limits of box constraints used in the fitting process. For example, 15% SWCCs without measured wet-end information were fitted with *α* values equal to a maximum value (100 m^−1^). Moreover, only 37% (1,714 out of 4,598 SWCCs) of SWCCs without wet-end information were assigned to the class of ‘good quality estimate’. We conclude that the limited number of measured data pairs and water content range imposes a large uncertainty of vG parameter values. But due to the scarcity of more complete measurements in many regions of the world, we must use such SWCCs as well that allow some general characterization of the unsaturated soil properties.

### Effect of climate on SWCCs

Soil hydraulic properties do not depend on soil texture alone but on soil structure as well, especially saturated hydraulic conductivity Ksat and the shape parameter *α* that is inversely related to the air entry value^10,18^. Soil-formation processes are particularly intense in tropical regions, it can be expected that SWCCs parameter values differ for tropical and other climatic regions^38^. For both clayey and loamy soils, we found larger *α* values for tropical regions. Similarly, Ottoni *et al*.^15^. also reported that soil hydraulic properties (vG parameters and Ksat) are different in the tropical compared to the temperate regions. Likewise, Gupta *et al*.^18^ illustrate that the PTFs developed using temperate Ksat measurements could not predict successfully tropical Ksat measurements. We hypothesize that this is due to the differences in the soil-forming processes that also determine the clay type and mineralogy. Tropical soils are often Oxisols rich inactive (non-swelling) clay minerals (kaolinite). In contrast to tropical soils, active (smectite) and moderately active clay minerals (illite) are the dominant clay minerals in other climate regions. These swelling clay minerals retain water within internal structures with strong capillary forces. Recently, Lehmann *et al*.^25^ revealed that the incorporation of clay-type informed PTFs could improve characterization of soil hydraulic and mechanical properties.

### Limitations of GSHP database

Some limitations need to be taken into account when using the GSHP database, as shortly detailed here. The database still lacks data from some regions (mainly Canada, and northern and western Australia). The database, in addition to SWCCs with ≥5 data pairs, also includes 9% SWCCs with 4 data pairs only (see spatial distribution of θ-*ψ* data pairs in Figure S4). For these SWCCs, the vG parameters are not well estimated because the measurements do not allow to properly capture the shape of the SWCC. 94% of SWCCs with 4 data pairs belong to the class without wet-end information. For some samples, we extracted the coordinates using Google Earth, and 12.3% SWCCs have no location accuracy information, hence, we advise using the coordinates with caution. Some estimated parameters were equal to the limits of the higher range of vG parameters but this represents only a limited portion (1% SWCCs attained the *n* value = 7 and 6.2% SWCCs obtained *α* = 100 m^−1^) of the total database. In the end, 11,705 SWCCs emerge as the good quality estimate after passing the profile likelihood test. Therefore, we recommend using these SWCCs with confidence, and other SWCCs should be used with care.

### Future applications of the GSHP database

The new GSHP database presents SWCC data from all continents and covers regions in Russia, which were so far not represented in currently available datasets and generally not included in PTF training and global mapping of soil hydraulic properties (note that there are still some gaps in the geographical representation of the data, especially data are lacking from Canada and northern and southern Australia). This global coverage is a great asset for the development of global PTFs for vG parameters. Remote sensing technology opens the doors to link measured hydraulic properties with global environmental data. For example, Gupta *et al*.^39^ linked Ksat with satellite-based maps of environmental covariates such as local information on vegetation, climate, and topography to generate a global map of Ksat with 1-km resolution. Likewise Chaney *et al*.^40^ developed the maps of soil hydraulic properties (POLARIS dataset) at 30 m resolution using remote sensing covariates for the United States using SSUGRO (Soil Survey Geographic) database. We recently used the GSHP dataset to generate highly resolved global maps of vG parameters. For that purpose, we applied machine learning approaches to relate vG parameters to soil and environmental covariates and predicted vG parameter values for all locations as a function of remote sensing information^41^.

## Supplementary information

Global Soil Hydraulic Properties dataset based on legacy site observations and robust parameterization

## Data Availability

All collected data and related soil characteristics are provided online for reference and are available at 10.5281/zenodo.5547338^37^. The R code used to create the GSHP database is available on Github (https://github.com/ETHZ-repositories/GSHP-database).
