# Supplementary material for: Global Soil Hydraulic Properties dataset based on legacy site observations and robust parameterization
Source: Sci Data. 2022 Jul 25;9:444. doi: 10.1038/s41597-022-01481-5 (PMC9314379; doi:10.1038/s41597-022-01481-5)
Supplement: Supplementary file 1 — Global Soil Hydraulic Properties dataset based on legacy site observations and robust parameterization [file 41597_2022_1481_MOESM1_ESM.pdf]

Supplementary Materials

Global Soil Hydraulic Properties dataset based on legacy site observations and robust parameterization

Surya Gupta[1], Andreas Papritz[1], Peter Lehmann[1], Tomislav Hengl[2,3], Sara Bonetti[4], Dani Or[1,5]

Affiliations

1. Soil and Terrestrial Environmental Physics, Department of Environmental Systems Science, ETH Zürich, Zürich, Switzerland

2. OpenGeoHub Foundation, Wageningen, the Netherlands

3. EnvirometriX, Wageningen, the Netherlands

4. Soil Physics and Land Management Group, Wageningen University, Wageningen, The Netherlands

5. Division of Hydrologic Sciences, Desert Research Institute, Reno, NV, USA

*corresponding author: Surya Gupta (surya.gupta@usys.ethz.ch)

List of Figures

S1 Examples of SWCCs that were either included or excluded from the GSHP database. a) and b) panels show SWCCs from the HYBRAS[1] dataset. a) panel shows that the water content at 0.001 m matric potential is lower than at 0.01 m matric potential. The SWCC was nevertheless included in the database as the increase of water content with increasing matric potential was less than 0.1 m^3/m^3. However, b) panel SWCC was removed as the difference exceeded 0.1 m^3/m^3. Similarly, SWCC c) was included and d) excluded (both SWCCs from Kool *et al.*[2]). 3

S2 Effect of constraining the estimated saturated water content θ_s for SWCCS with missing wet-end measurements. a) vG models fitted without PTF-derived constraint for θ_s. b) vG models fitted with box constraints for θ_s that was derived from the linear regression PTF for θ_s. The numbers in panel b) show the upper and lower limits of 95%-prediction intervals for θ_s that were used as as box constraints for this parameter. The black dotted lines indicate the field capacity (water contents at 1 m and 3.3 m) and permanent wilting point (water content at 150 m). 4

S3 Locations of SWCCs along with information whether saturated hydraulic conductivity Ksat data is available (8,675 samples from 1,279 locations). 4

S4 Locations of SWCCs classified based on the number of SWCCs data pairs as shown in Table 3 in the main text. . 4

List of Tables

S1 Number of samples (N) assigned to each spatial accuracy class. NA are samples without information on spatial accuracy. The location accuracy is in meters. 5

S2 Illustrative examples for the effect of constraining the estimated saturated water content θ_s for SWCCS with missing wet-end measurements on the estimated vG parameters (α (m^{-1}), n, θ_r (m^3/m^3) and θ_s (m^3/m^3) see Figure S2). Three SWCCs were randomly selected from the WOSIS dataset with different soil textures (layer_id 598007, 655174, 656392 in GSHP database). The smallest measured matric potential of these SWCCs was 1 m. The SWCC fitting without wet-end leads to different vG parameters. θ_s is approxmiately equal to the water content at 1 m matric potential when fitted without imputed θ_s. 5

S3 Details on parameter constraints used for estimation of SWCC parameters. YW and NW stand for SWCCs with and without wet-end information, respectively, while YD and ND stand for SWCCs with and without dry-end information, respectively. NPTFU stands for ‘no PTF-based box constraint used’ whereas PTFU stands for ‘PTF-based box constraint used’. Note that for α and n a range of 0-100 (m^{-1}) and 1-7, respectively, were used for all SWCCs. 5

S4 Varianance-covariance matrix and residual standard error of Model1. 6

S5 Varianance-covariance matrix and residual standard error of Model2. 6

S6 Total number of SWCCs (N) from each continent. 6

S7 Statistics of number of soil profiles, samples, and depths per location. 6

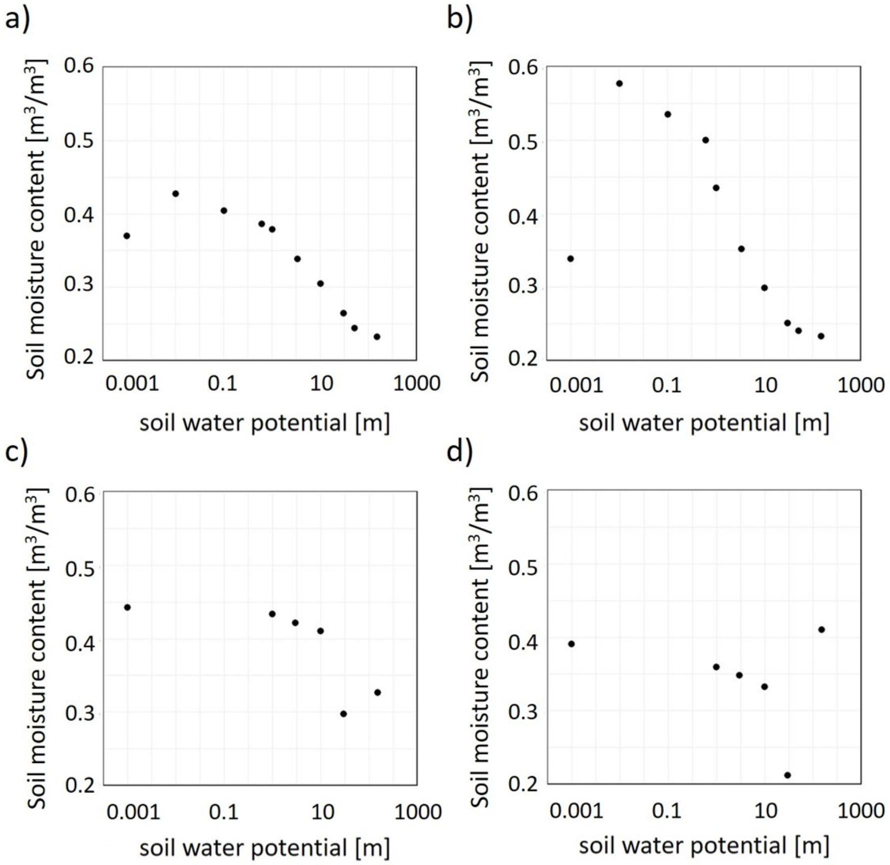

Figure S1. Examples of SWCCs that were either included or excluded from the GSHP database. a) and b) panels show SWCCs from the HYBRAS[1] dataset. a) panel shows that the water content at 0.001 m matric potential is lower than at 0.01 m matric potential. The SWCC was nevertheless included in the database as the increase of water content with increasing matric potential was less than 0.1 m^3/m^3. However, b) panel SWCC was removed as the difference exceeded 0.1 m^3/m^3. Similarly, SWCC c) was included and d) excluded (both SWCCs from Kool *et al.*[2]).

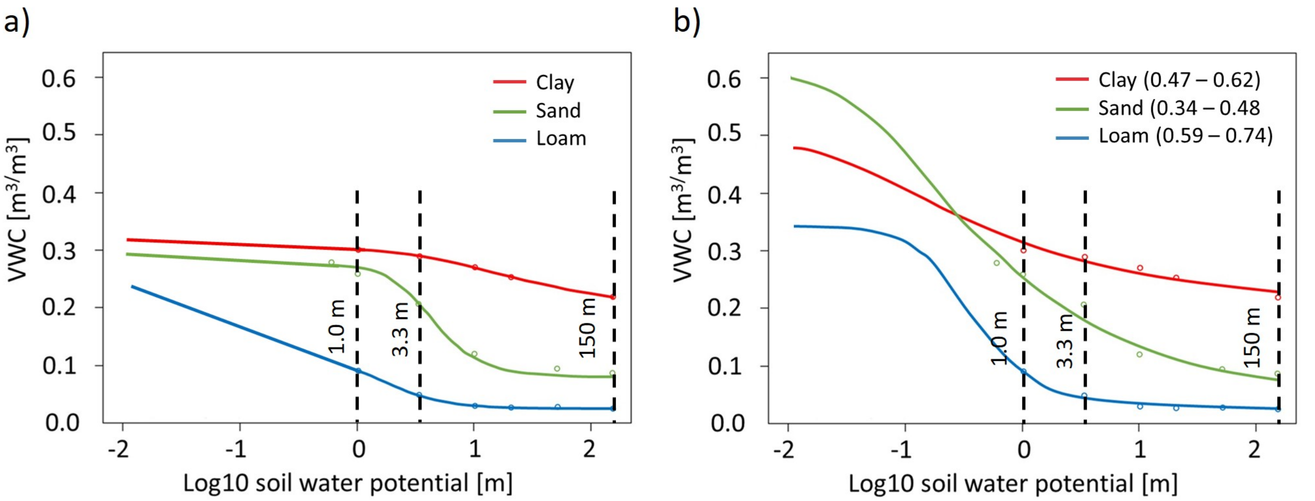

Figure S2. Effect of constraining the estimated saturated water content θ_s for SWCCS with missing wet-end measurements. a) vG models fitted without PTF-derived constraint for θ_s. b) vG models fitted with box constraints for θ_s that was derived from the linear regression PTF for θ_s. The numbers in panel b) show the upper and lower limits of 95%-prediction intervals for θ_s that were used as as box constraints for this parameter. The black dotted lines indicate the field capacity (water contents at 1 m and 3.3 m) and permanent wilting point (water content at 150 m).

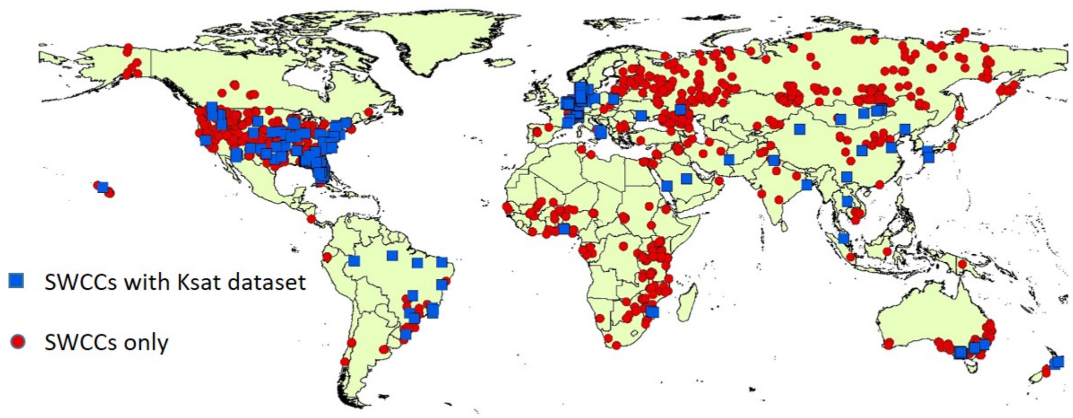

Figure S3. Locations of SWCCs along with information whether saturated hydraulic conductivity Ksat data is available (8,675 samples from 1,279 locations).

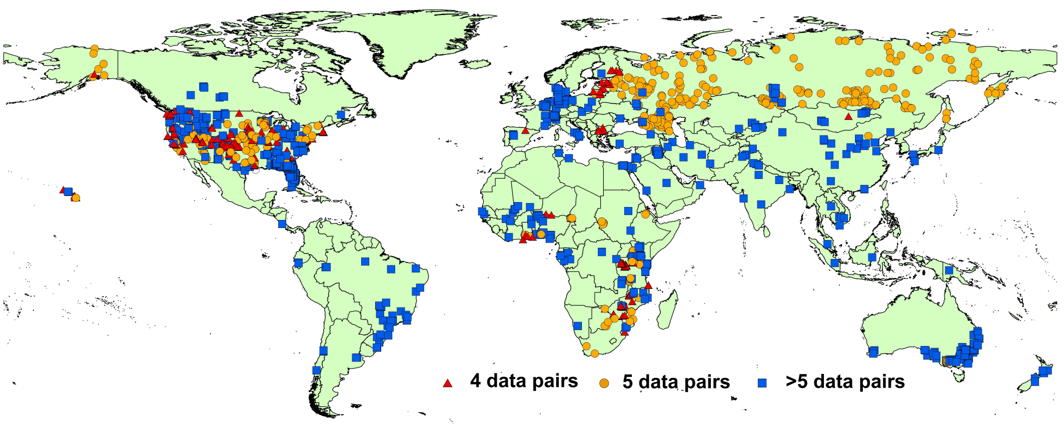

Figure S4. Locations of SWCCs classified based on the number of SWCCs data pairs as shown in Table 3 in the main text.

Table S1. Number of samples (N) assigned to each spatial accuracy class. NA are samples without information on spatial accuracy. The location accuracy is in meters.

s.no	max-min location accuracy	N
1	0-100	10,373
2	100-250	389
3	250-500	66
4	500-1000	150
5	1000-5000	2,157
6	5000-10000	179
7	10000-NA	55
8	NA	1,890

Table S2. Illustrative examples for the effect of constraining the estimated saturated water content θ_s for SWCCS with missing wet-end measurements on the estimated vG parameters (α (m^{-1}), n, θ_r (m^3/m^3) and θ_s (m^3/m^3) see Figure S2). Three SWCCs were randomly selected from the WOSIS dataset with different soil textures (layer_id 598007, 655174, 656392 in GSHP database). The smallest measured matric potential of these SWCCs was 1 m. The SWCC fitting without wet-end leads to different vG parameters. θ_s is approxmiately equal to the water content at 1 m matric potential when fitted without imputed θ_s.

Soil type	parameters estimated without box-constraint for θ_s				θ_s at 1 m MP	parameters estimated with box-constraints for θ_s			
	α	n	θ_r	θ_s		α	n	θ_r	θ_s
Clay	2.30	1.05	0.00	0.34	0.31	100	1.15	0.18	0.48
Loam	0.30	2.56	0.09	0.27	0.26	16.5	1.34	0.03	0.59
Sand	0.80	2.29	0.02	0.11	0.09	5.49	1.90	0.02	0.34

Table S3. Details on parameter constraints used for estimation of SWCC parameters. YW and NW stand for SWCCs with and without wet-end information, respectively, while YD and ND stand for SWCCs with and without dry-end information, respectively. NPTFU stands for 'no PTF-based box constraint used' whereas PTFU stands for 'PTF-based box constraint used'. Note that for α and n a range of 0-100 (m^{-1}) and 1-7, respectively, were used for all SWCCs.

SWCCs	SWCC classes	number of SWCCs	Parameterization of vG parameters	
			θ_s (m^3/m^3)	θ_r (m^3/m^3)
SWCCs	YWYD	10,301	NPTFU	NPTFU
	YWND	333	NPTFU	PTFU
	NWYD	4,598	PTFU	NPTFU
	NWND	27	PTFU	PTFU

Table S4. Varianance-covariance matrix and residual standard error of Model1.

	Intercept	BD	Sand	Clay	Tropical climate
Intercept	7.3637e-05	-4.0378e-05	-7.3033e-08	-4.8954e-07	-4.5667e-06
BD	-4.0378e-05	2.6645e-05	-4.8126e-08	1.8556e-07	4.0320e-06
Sand	-7.3033e-08	-4.8126e-08	2.5317e-09	1.7842e-09	-4.9783e-08
Clay	-4.8954e-07	1.8556e-07	1.7842e-09	6.0044e-09	-1.7017e-08
Tropical climate	-4.5667e-06	4.0320e-06	-4.9783e-08	-1.7017e-08	9.1626e-06
Residual standard error			0.04031		

Table S5. Varianance-covariance matrix and residual standard error of Model2.

	Intercept	BD	Tropical climate
Intercept	4.2762e-05	-2.9529e-05	-6.7082e-06
BD	-2.9529e-05	2.1205e-05	3.6384e-06
Tropical climate	-6.7082e-06	3.6384e-06	9.4614e-06
Residual standard error		0.04204	

Table S6. Total number of SWCCs (N) from each continent.

Continent	N	Continent	N
North America	10,408	South America	858
Europe	1,130	Australia/Oceania	832
Africa	943	Antarctica	17
Asia	968		

Table S7. Statistics of number of soil profiles, samples, and depths per location.

No. of locations	No of profiles	No. of locations	No of samples	No. of locations	Number of depths
214	0	259	1	32	0
2390	1	1,418	2-5	287	1
60	2-5	916	6-10	1,447	2-5
17	6-10	109	>10	876	6-10
21	>10	—	—	60	>10

References

1. Ottoni, M. V., Ottoni Filho, T. B., Schaap, M. G., Lopes-Assad, M. L. R. & Rotunno Filho, O. C. Hydrophysical database for brazilian soils (hybras) and pedotransfer functions for water retention. *Vadose Zone J.* **17** (2018).

2. Kool, J., Albrecht, K. A., Parker, J., Baker, J. *et al.* Physical and chemical characterization of the groseclose soil mapping unit. Tech. Rep., Virginia Agricultural Experiment Station (1986).
